# Significantly Delayed Medium-Latency Response of the Stretch Reflex in Delayed-Onset Muscle Soreness of the Quadriceps Femoris Muscles Is Indicative of Sensory Neuronal Microdamage

**DOI:** 10.3390/jfmk7020043

**Published:** 2022-05-27

**Authors:** Balázs Sonkodi, Ádám Hegedűs, Bence Kopper, István Berkes

**Affiliations:** 1 Department of Health Sciences and Sport Medicine, Hungarian University of Sport Science, 1123 Budapest, Hungary; berkesdr@gmail.com; 2 Faculty of Kinesiology, Hungarian University of Sport Science, 1123 Budapest, Hungary; adam.hegedus.tf@gmail.com (Á.H.); kopper.tf@gmail.com (B.K.)

**Keywords:** medium-latency response of the stretch reflex, delayed-onset muscle soreness, non-contact injury, Piezo2 ion channel, Type Ia fiber, proprioception, muscle spindle, electromyography

## Abstract

Unaccustomed or strenuous eccentric exercise is known to cause delayed-onset muscle soreness. A recent hypothesis postulated that mechano-energetic microinjury of the primary afferent sensory neuron terminals in the muscle spindles, namely a transient Piezo2 channelopathy, could be the critical cause of delayed-onset muscle soreness in the form of a bi-phasic non-contact injury mechanism. This theory includes that this microlesion could delay the medium-latency response of the stretch reflex. Our aim with this study was to investigate this hypothesis. According to our knowledge, no study has examined the effect of delayed-onset muscle soreness on the medium-latency response of the stretch reflex. Our findings demonstrated that a significant delay in the medium-latency stretch reflex could be observed right after a multi-stage fitness test in the quadriceps femoris muscles of Hungarian professional handball players who consequently experienced delayed-onset muscle soreness. The long-latency stretch reflex and most likely short-latency stretch reflex were unaffected by delayed-onset muscle soreness in our study, which is in line with earlier findings. We translate these findings as indicative of proprioceptive Type Ia terminal microdamage in the muscle spindle in line with the aforementioned new acute non-contact compression axonopathy theory of delayed-onset muscles soreness.

## 1. Introduction

Unaccustomed and strenuous exercise comprising repetitive, fatiguing eccentric contractions often induces delayed-onset muscle soreness (DOMS). DOMS is defined as a delayed onset of soreness, muscle stiffness, swelling, loss of force-generating capacity, reduced joint range of motion and diminished proprioceptive function [1]. The pain of DOMS is not felt for about 8 h and peaks 1 or 2 days later [2].

However, the exact mechanism of DOMS is still far from entirely explored. Several theories are currently running, such as lactic acid, muscle spasm, inflammation, connective tissue damage, muscle damage, enzyme efflux and, most recently, proprioceptive terminal microdamage or transient Piezo2 channelopathy theory [3,4,5], but no single theory or factor has answered the problem entirely. It is notable that DOMS is not the same as pain experienced during or immediately after exercise [6]. DOMS could be induced even without muscle damage [7]; for instance, vibration could evoke it [8]. Additionally, exercise-induced muscle damage could exist without DOMS, and earlier, it was viewed that DOMS inducement is independent of inflammation [9], or at least that it is not essential for DOMS [10].

On the contrary, a newly published acute non-contact compression axonopathy hypothesis of DOMS postulates that the critical cause could be the mechano-energetic microdamage of the proprioceptive neuron terminals in the muscle spindles due to a cognitive-demand-derived acute stress response on top of unaccustomed or strenuous repetitive eccentric contractions [4]. Later, it was hypothesized that the locus of this causal neuron terminal microdamage is on Piezo2 ion channels, in the form of a transient Piezo2 channelopathy [5]. Moreover, recent hypotheses suggest that delayed-onset muscle soreness is an analogous bi-phasic non-contact injury mechanism, such as non-contact anterior cruciate ligament (ACL) injury and post-orgasmic illness syndrome (POIS), where the primary microinjury is a proprioceptive Piezo2 channelopathy and the secondary injury is harsher tissue damage due to the loss of proprioception [4,5,11]. Indeed, Piezo2 has been shown to be the principal mechano-transduction channel for proprioception and contributes to vibration sensing and touch discrimination [12,13,14]. Notably, Morgan et al. and Hody et al. described the dichotomous injury mechanism of DOMS, and Weerakkody et al. already implicated the contribution of large fiber proprioceptive neurons of the muscle spindles in DOMS [8,15,16]. Torres et al. [17] demonstrated in their study that DOMS reduces proprioception immediately after DOMS-inducing exercise [17]. Moreover, they also suggested that the locus of impairment is in the muscle spindle; however, they blamed the intrafusal muscles for the impairment [17].

Bennet et al. put forward a novel concept of neuronal terminal lesions, called TAD lesions, that could be learnt from chemotherapy and do not come with a classical Wallerian degeneration [18]. Kouzaki et al. showed that eccentric-exercise-induced muscle damage resulted in an increased latency of M-wave and even implicated reversible motoneuronal damage but ruled out muscle spindle origin [19]. However, Vincent et al. demonstrated as a consequence of platinum analogue chemotherapy that it could cause complex Type Ia proprioceptive impairment and that this lesion could occur in an acute and chronic manner as well [20,21]. In addition, Alvarez et al. and Bullinger et al. showed chronic central synaptic disconnection of proprioceptors from motoneurons after nerve injury, and this phenomenon is dedicated to the loss of vesicular glutamate transporter (VGLUT) 1/Ia synapses on motoneurons [22,23]. Nevertheless, the current authors suggest that the muscle spindle origin of increased M-wave latency on motoneurons after eccentric-exercise-induced muscle damage should not be excluded. They even proposed that the acutely or reversibly impaired muscle spindle derived Type Ia proprioceptive terminals and the resultant synaptic disconnection on motoneurons is to blame for the increased M-wave latency and the suggested reversible motoneuronal damage in DOMS.

Accordingly, it has been theorized that a minor alteration of the stretch reflex in the form of delayed latency of medium-latency response (MLR) could be the result of the mechano-energetic Piezo2 microlesion of proprioceptive sensory terminals in DOMS due to the transient central disconnection of monosynaptic Type Ia synapses on motoneurons and a compensatory switch to polysynaptic Type II synapses [5,24]. There are three components of the stretch reflex: short-latency (dominantly Type Ia afferent mediated), medium-latency (most likely dominantly Type II afferent mediated), and long-latency (presumably transcortical or subcortical) responses [25,26,27,28,29,30,31,32,33,34]. 

The aim of our current study was to investigate the effect of DOMS on the latency of MLR, because we found no study in the literature examining MLR of the stretch reflex affected by DOMS. It is worth noting that both the short-latency response (SLR) and long-latency response (LLR) times of the stretch reflex were shown to be unaffected by DOMS [35]. Correspondingly, we were seeking to find out how an acute fatiguing task changes the time characteristics of the MLR based on the SLR. We chose handball players for our study because they are often exposed to landing, side-cutting, or deceleration with heavy involvement of eccentric quadriceps forces that could lead to the aforementioned non-contact injuries [36]. Moreover, we opted for a scheduling of our study when the candidate athletes returned from an almost-two-month summer recess in order to attain the unaccustomed status in our acute fatiguing exercise protocol.

## 2. Methods

### 2.1. Participants

Nine female professional handball players (mean ± SD age: 24.11 ± 3.72) participated in this study. Before the experiment, they were informed about the protocol and signed a written consent. The Ethical Committee of the Hungarian University of Sport Science (TE-KEB/26/2021) approved the experimental protocol.

### 2.2. Procedures

The protocol consisted of two surface electromyography (sEMG) measurements and a multi-stage fitness test (MSFT) between the two. EMG activity of the vastus medialis (VMO) and the rectus femoris (REC) were recorded during the two measurements. Prior to the first sEMG measurement, participants’ legs were prepared, to obtain the most accurate EMG signal and best signal–noise ratio possible. The hair was removed with a razor, then the mortified epithelium was sanded with sandpaper; lastly, we washed the skin with alcohol [37]. Two unipolar Ambu Blue Sensor “N” electrodes were placed onto the muscles in union with the SENIAM electrode preparation and placement protocol recommendations (the electrode center distance was 20 mm), and the ground electrode was placed onto the patella. To establish similar measurement parameters for the before–after data collection, electrodes remained in place on the participants during the fatiguing protocol. Noraxon MyoResearch Master Edition version 1.08.27 software (Noraxon, Scottsdale, Arizona 85260, USA 2016) was used for detection and data processing of the EMG signals. The dominant leg of the handball players was measured.

One of the examiners handled the sEMG laptop, while the other’s responsibility was to evoke the stretch reflex. The participants had to initiate a seating position with electrodes placed on their dominant leg in 90-degree flexion, and their task was to generate MVC (maximal voluntary contraction) in the quadriceps, while the assistant pulled the elastic band, which was placed around the ankle of the dominant leg in order to maintain this 90-degree knee flexion. The participants were instructed to aim for MVC during contractions, when the examiner would suddenly release the elastic band, and their job was to return to the 90-degree flexion as fast as they possibly could. Two measurements were recorded in all cases: one before and one instantly after the fatiguing 20 min MSFT was finished.

### 2.3. Exercise Protocol

To execute measurements when the possibility of DOMS occurrence is high and the athletes are in a controlled environment, the universally accepted and globally used MSFT test was chosen. As the MSFT test consists of phases of rapid acceleration and deceleration, it is an ideal test for the purpose of the study. The MSFT is a test commonly used to measure aerobic fitness. The MSFT consists of continuous running between two lines 20 m apart in time to recorded beeps. The speed at the start of the test is slow. After one minute, an increase in speed happens, and the time between beeps decreases. Each minute, the time between beeps decreases, so a level consists of 1 min. If the line is reached before the beep sounds, the subject must wait until the beep sounds before continuing. If the line is not reached before the beep sounds, the subject is given a warning, and after the second warning, the subject is eliminated.

### 2.4. Electromyography

For processing sEMG data, Noraxon MyoResearch Master Edition version 1.08.27 software was used. The frequency range used a low- and high-band filter module under 5 Hz, over 350 Hz were cut, 50–60 Hz frequency domains were filtered, and the filtered data were used for smoothening in the built-in smoothening module of the Noraxon software.

### 2.5. Statistics

As we had a limited sample size, and for the purpose of selecting the adequate statistical procedure, Shapiro Wilk’s W test was used to check the normal distribution of the data. Only the VMO before the MSFT was not normally distributed, so for comparing the VMO data, the Wilcoxon nonparametric test was used, while for comparing the REC data, a dependent *t*-test was used. To further support the results of this study, we have calculated and included the effect size (ES-Cohen’s d) value and also calculated the statistical power for the significant differences. StatSoft STATISTICA 13.2 was used for the statistical analysis; significance level was set at *p*  <  0.05.

### 2.6. Muscle Soreness Questionnaire 

We sent out an electronic statement form one day after the MSFT to the professional handball players to declare whether they experienced DOMS after the MSFT. As all the participants were high-level professional athletes, the subjective questionnaire was based on their knowledge concerning their previous DOMS experience. 

## 3. Results

### 3.1. EMG Activity of the Observed Muscles 

**Figure 1:**
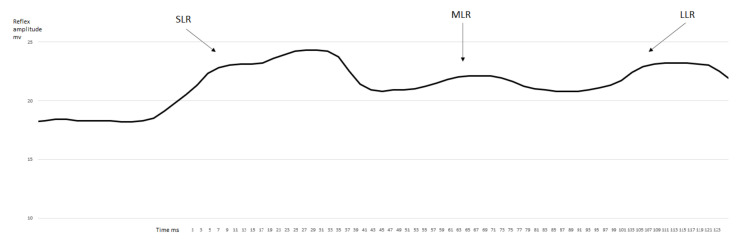
Representative rectified, filtered, and smoothened curve of the EMG activity for the rectus femoris muscle before the fatiguing protocol.

**Figure 2:**
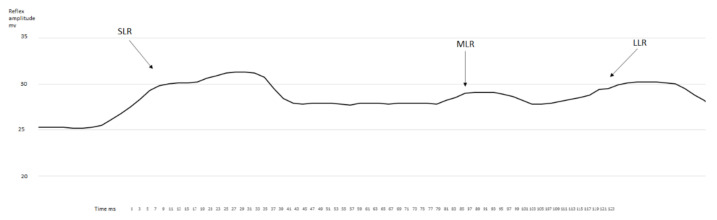
Representative rectified, filtered, and smoothened curve of the EMG activity for the rectus femoris muscle after the fatiguing protocol.

**Figure 3:**
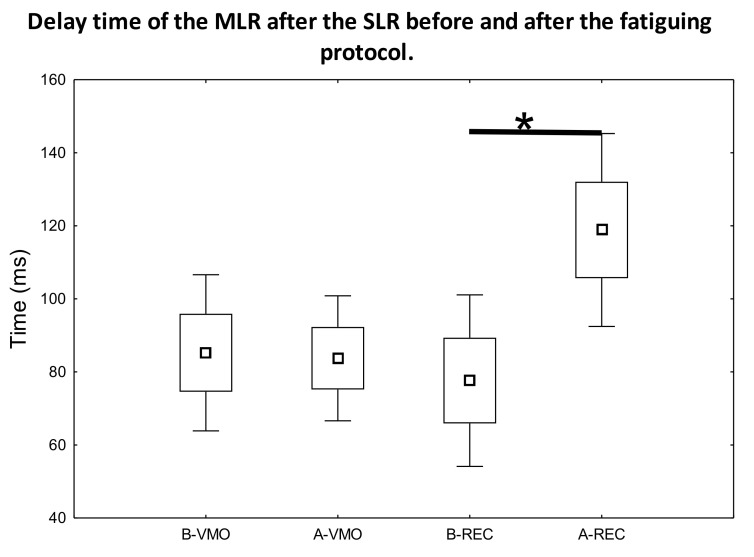
Delay time (ms) of the MLR after the SLR before and after the fatiguing protocol (B-VMO, B-REC measurement data taken on the muscles before fatiguing protocol; A-VMO, A-REC measurement data after fatiguing protocol). Box: mean ± SE; whiskers: mean ± 2SE. * indicates significant difference between B-REC and A-REC (*p* < 0.05). No significant difference could be observed between B-VMO and A-VMO (*p* > 0.05).

**Figure 4:**
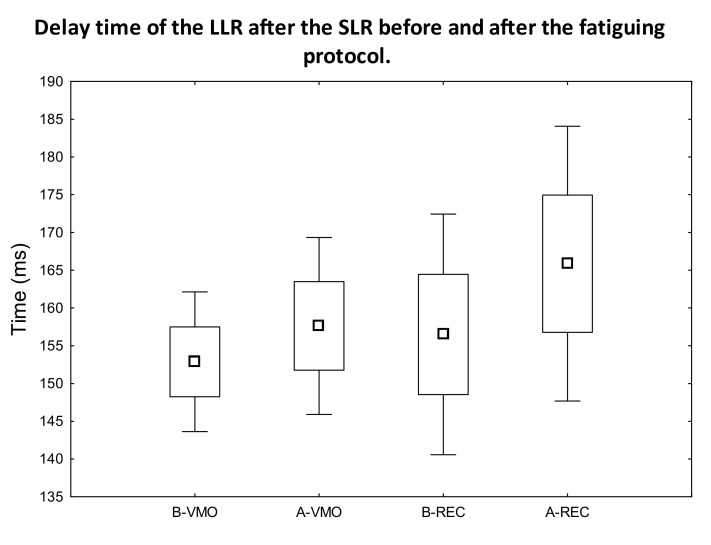
Delay time of the LLR after the SLR before and after the fatiguing protocol. Box: mean ± SE; whiskers: mean ± 2SE. No significant difference could be observed between B-VMO and A-VMO and between B-REC and A-REC, respectively (*p* > 0.05).

After the evaluation of the obtained data determined from the measured sEMG signal (Figure 1 and Figure 2 representative curves for samples of data measured on different individuals), the SLR, MLR, and LLR times were determined as the unambiguously definable local maximum peak values in the sEMG-time figures. Subsequently, we compared the latency-time values between the SLR-MLR and SLR-LLR for the measured muscles. A statistically significant increase was only observed in the latency of MLR (Figure 3) compared with the SLR of the rectus femoris muscle after the fatiguing protocol (*p* = 0.017, ES = 1.16, power = 0.56). In no other comparisons for the VAC and REC muscles between the before–after latency values could a significant difference be observed (Figure 4).

### 3.2. Muscle Soreness Questionnaire 

**Figure 5:**
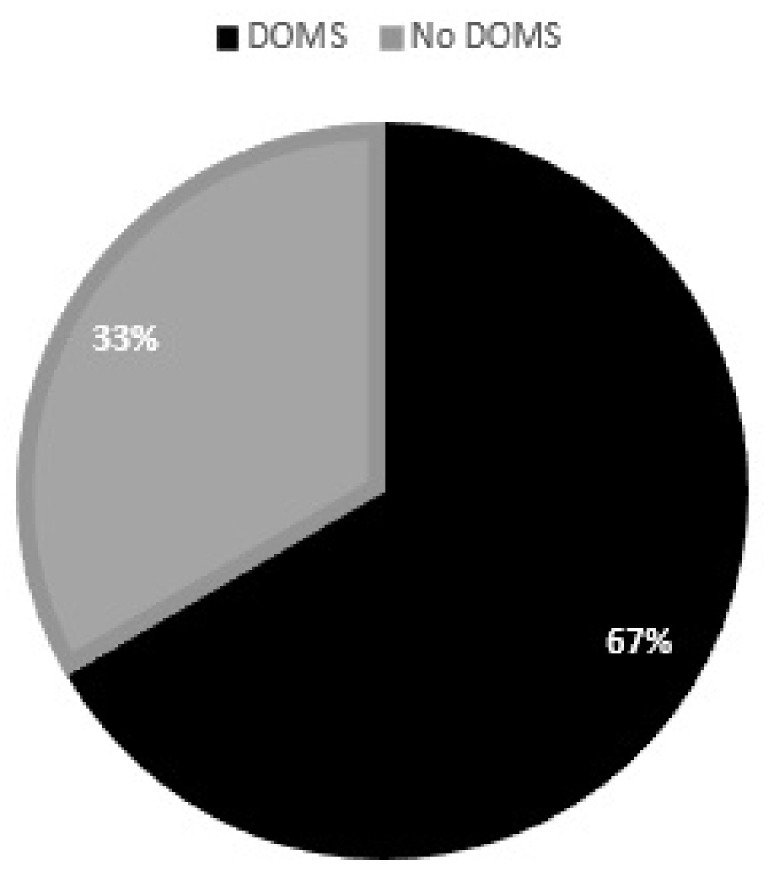
Percentage of handball players with DOMS after the fatiguing protocol.

All players sent back the statement form with their declaration within 48 h. After filling out these questionnaires, six of the nine participants reported DOMS after the fatiguing protocol (Figure 5).

## 4. Discussion

The results of our study substantiate our hypothesis that DOMS-inducing exercise delays the latency of the MLR (Figure 3). Moreover, the delayed latency of MLR supports the theory of muscle-spindle involvement and proprioceptive neuronal microdamage in DOMS [4]. Furthermore, this muscle-spindle-involved impairment can be measured by EMG right after DOMS-inducing exercise and hours before the onset of mechanical hyperalgesia. It is also notable that muscle soreness starts at about 8 h after DOMS-inducing exercise [2], so evidence of neuronal impairment could be detected earlier—indeed, right after DOMS-inducing exercise. Our results also indicate that LLR is unaffected by DOMS (Figure 4), which correlates with the earlier findings of Hjortskov et al. [35] and with the hypothesis of Sonkodi [24]. In addition, based on previous studies, the SLR seems to also be unaffected in terms of delay; consequently, we used it as a reference point in our study. 

Eccentric contractions increase when muscles are fatigued [38]. Proske et al. demonstrated that repetitive eccentric contractions damage proprioception [39]. The acute non-contact compression proprioceptive axonopathy theory of DOMS puts forward that these repetitive unaccustomed or strenuous eccentric contractions could cause microdamage to the proprioceptive axon terminals in the muscle spindles [4,24]. This terminal microinjury is proposed to be terminal arbor degeneration (TAD) such as mechano-energetic lesions on the peripheral ends of these pseudo-unipolar proprioceptive somatosensory fibers [4]. Paclitaxel-based chemotherapy also exhibits this type of terminal sensory TAD lesion, evolves after a dose-dependent and threshold-driven manner, and is not associated with classical Wallerian axonal degeneration [18]. Furthermore, it has been demonstrated that oxaliplatin treatment, another chemotherapeutic agent, abolished the static encoding of the muscle position, while the dynamic change components were left unaffected [21].

Correspondingly, and in line with the acute compression proprioceptive axonopathy theory, it has been hypothesized that DOMS alters only the static-phase firing sensory encoding of the mostly unaffected stretch reflex of the preprogrammed postural control [24,40]. This minor alteration is presumably due to the exchange of monosynaptic static-phase firing sensory encoding of the stretch reflex induced by disconnected monosynaptic Type Ia afferents to Type II polysynaptic afferent ones [24]. Both Type Ia and Type II sensory fibers contribute to static-phase firing sensory encoding of the stretch reflex, but only Type Ia transcends dynamic encoding. Accordingly, it is theorized that the encoding of the dynamic-change sensory component is barely affected, as it could be observed in platinum-analogue chemotherapy [21,40]. Therefore, no delay in the latency of SLR was expected due to the effect of DOMS. 

The actual microdamage that could lead to this Type Ia impairment is suggested to be energy-depleted-mitochondria-induced dysfunctional glutamate vesicular release and Piezo2 channelopathy [5]. It is important to highlight that Piezo2 ion channels are the principal mechanotransduction channels for proprioception [12]. The evoked secondary polysynaptic compensatory pathway on the spinal dorsal horn, as a consequence of the microdamage, is suggested to be preprogrammed [11,24]. This exchange of static monosynaptic Type Ia afferent connection to Type II polysynaptic afferent ones has been proposed to be reflected in the delayed latency of the MLR of the affected stretch reflex [24,40]. It is noticeable that the conduction velocity of Type II sensory fibers is slower than Type Ia fibers and polysynaptic signaling is slower than monosynaptic signaling. Moreover, this compensatory exchange to polysynaptic signaling is highly consuming neuro-energetically within the proprioceptive system, which is suggested to have resource limitations [11,24]. Furthermore, numerous studies point toward MLR being presumably dominantly Type-II-afferent-mediated [25,26,27,28,29,30,31,32,33,34]. Moreover, it is important to note that fatigue by itself, which is, per definition, implicit in DOMS, decreases and does not delay the amplitude of MLR [41,42]. Accordingly, the authors translate the delayed latency of MLR as indicative of proprioceptive neuronal microdamage in the muscle spindle. 

The above findings could be indicative of the acute compression proprioceptive axonopathy theory of DOMS, in which the hypothesized microinjury of the Type Ia afferent terminals is due to Piezo2 ion channel impairment [5]. The inactivation of Piezo2 channels in a hyperexcited state is a physiological response, and it is considered to be within homeostasis [43,44]. However, Sonkodi et al. theorized that the inactivated Piezo2 channels could incur microdamage under an acute stress response (ASR) and could become leaky to unbalanced, mainly due to Ca^2+^, subthreshold currents, and glutamate [5,45]. Glutamate is a key and also fast neurotransmitter under pathological conditions of the nervous system, as in the case of traumatic injuries [46,47]. Part of the acute compression proprioceptive axonopathy theory of DOMS that unaccustomed or strenuous eccentric contractions and an ASR on top of it could induce energy depletion at the hyperexcited Type Ia terminals and, as a result, dysfunctional mitochondria could impair glutamate vesicular release, leading to glutamate spillover [5]. The 2–3 day symptoms of POIS were attributed to this type of Piezo2 channelopathy without C-fiber contribution and additional tissue damage [5]. Indeed, C-fiber contribution is essential from eccentric exercised muscles in DOMS in order to provide the slow temporal summation [48,49], but must involve other factors than muscle damage [10], and that is suggested to be the microdamage of the Type Ia terminal, or, more precisely, the Piezo2 channelopathy [4,5]. Therefore, Sonkodi et al. postulated in their theory that the secondary phase of DOMS with harsher tissue damage with C-fiber contribution is needed for the seven-day-long acute compression axonopathy symptoms beyond the Piezo2 microdamage, as it is needed in the seven-day-long POIS symptoms as well [4,5]. 

In summary, the authors of this study suggest that a Piezo2 channelopathy on Type Ia sensory terminals in the muscle spindle could be responsible for the compensatory exchange of static-phase firing sensory encoding of the stretch reflex from Type Ia to Type II afferents, and, as a result, for the delayed latency of the MLR (Figure 3). The impaired static-phase firing sensory encoding of the stretch reflex in the form of delayed-latency MLR could also explain the impaired proprioception symptom of DOMS. It is important to emphasize that the world delay in the delayed-latency of MLR and delayed-onset muscle soreness are not equivalent. In fact, the delayed latency of MLR refers to the primary microinjury of DOMS, while the delayed pain sensation refers more to the secondary damage phase of DOMS. However, the secondary harsher tissue injury is the direct result of the impaired proprioception. Correspondingly, this current study demonstrated that DOMS is associated with a delayed latency of MLR. However, it is important to note that proprioceptive Piezo2 microinjury alone could cause a delayed latency of MLR without DOMS involvement due to the absence of the secondary injury phase. In this case, that is when damaging eccentric contractions are discontinued right after the primary injury without secondary harsher tissue damage; it is a pain free condition because there is no C fiber contribution or slow temporal summation of pain sensation [5].

It should be noted that reduced proprioception, which is one consequence of DOMS, could increase the risk of sport injuries based on common consent [17]. Furthermore, research is emerging in support of DOMS-associated neurodamage and neuroinflammation in DOMS; for instance, Borghi et al. showed recently that intense acute DOMS-inducing swimming causes spinal-cord neuroinflammation [50]. The current authors translate these findings that spinal-cord neuroinflammation in DOMS could be the consequence of the Piezo2 channelopathy and the resultant central synaptic disconnection of the same proprioceptor from motor neurons.

## 5. Limitations

One obvious limitation of our study is that the measured sample size was low, as the calculated moderate power value suggests. We conducted our data collection in the middle of the SARS-CoV-2 pandemic; therefore, the number of recruitable participants was limited. Thus, our results could be considered as a preliminary verification of a theory study, and further investigation is recommended with greater sample size. However, the calculated very high effect-size value indicates that the intervention in the fatiguing protocol indeed had an effect on the neuromuscular system, which substantially supports our hypothesis.

Moreover, a primary definite DOMS-inducing protocol was not set by the current researchers, which could also be a limitation. We did not want to intervene in the training regime of these professional athletes during the SARS-CoV-2 pandemic, but we used the opportunity to make EMG measurements prior to and after the MSFT ordered by the coach of this Hungarian professional female handball team. 

A further limitation could be that DOMS was confirmed only by a questionnaire that was sent to the handball players electronically two days after the MSFT. Re-inviting the professional handball players for more objective DOMS measurement was problematic due to their tight schedule and also the SARS-CoV-2 pandemic. 

## 6. Conclusions

Our result demonstrates that potentially DOMS-inducing MSFT indeed delays the latency response of MLR in the quadriceps femoris muscles (Figure 3) of Hungarian female professional handball players immediately after the exercise protocol. Furthermore, our result could theoretically explain the earlier findings, namely the reduction in proprioception immediately after DOMS-inducing exercise. Moreover, the LLR and most likely SLR were unaffected in terms of delay (Figure 4) by DOMS in our study, which is also in line with earlier findings. These results add to our understanding of the hypothetical proprioceptive Type Ia afferent mechano-energetic microdamage, which could be the critical cause of DOMS. 

## Data Availability

The data presented in this study are available on request from the corresponding author.

## References

[1] Clarkson P.M., Nosaka K., Braun B. (1992). Muscle function after exercise-induced muscle damage and rapid adaptation. Med. Sci. Sports Exerc..

[2] Newham D.J. (1988). The consequences of eccentric contractions and their relationship to delayed onset muscle pain. Eur. J. Appl. Physiol. Occup. Physiol..

[3] Cheung K., Hume P., Maxwell L. (2003). Delayed onset muscle soreness: Treatment strategies and performance factors. Sports Med..

[4] Sonkodi B., Berkes I., Koltai E. (2020). Have We Looked in the Wrong Direction for More Than 100 Years? Delayed Onset Muscle Soreness Is, in Fact, Neural Microdamage Rather Than Muscle Damage. Antioxidants.

[5] Sonkodi B., Kopa Z., Nyirady P. (2021). Post Orgasmic Illness Syndrome (POIS) and Delayed Onset Muscle Soreness (DOMS): Do They Have Anything in Common?. Cells.

[6] Miles M.P., Clarkson P.M. (1994). Exercise-induced muscle pain, soreness, and cramps. J. Sports Med. Phys. Fit..

[7] Hayashi K., Abe M., Yamanaka A., Mizumura K., Taguchi T. (2015). Degenerative histological alteration is not required for the induction of muscular mechanical hyperalgesia after lengthening contraction in rats. J. Physiol. Sci..

[8] Weerakkody N.S., Percival P., Hickey M.W., Morgan D.L., Gregory J.E., Canny B.J., Proske U. (2003). Effects of local pressure and vibration on muscle pain from eccentric exercise and hypertonic saline. Pain.

[9] Semark A., Noakes T.D., St Clair Gibson A., Lambert M.I. (1999). The effect of a prophylactic dose of flurbiprofen on muscle soreness and sprinting performance in trained subjects. J. Sports Sci..

[10] Mizumura K., Taguchi T. (2016). Delayed onset muscle soreness: Involvement of neurotrophic factors. J. Physiol. Sci..

[11] Sonkodi B., Bardoni R., Hangody L., Radák Z., Berkes I. (2021). Does Compression Sensory Axonopathy in the Proximal Tibia Contribute to Noncontact Anterior Cruciate Ligament Injury in a Causative Way?—A New Theory for the Injury Mechanism. Life.

[12] Woo S.H., Lukacs V., de Nooij J.C., Zaytseva D., Criddle C.R., Francisco A., Jessell T.M., Wilkinson K.A., Patapoutian A. (2015). Piezo2 is the principal mechanotransduction channel for proprioception. Nat. Neurosci..

[13] Chesler A.T., Szczot M., Bharucha-Goebel D., Ceko M., Donkervoort S., Laubacher C., Hayes L.H., Alter K., Zampieri C., Stanley C. (2016). The Role of PIEZO2 in Human Mechanosensation. N. Engl. J. Med..

[14] Ranade S.S., Woo S.H., Dubin A.E., Moshourab R.A., Wetzel C., Petrus M., Mathur J., Begay V., Coste B., Mainquist J. (2014). Piezo2 is the major transducer of mechanical forces for touch sensation in mice. Nature.

[15] Hody S., Croisier J.L., Bury T., Rogister B., Leprince P. (2019). Eccentric Muscle Contractions: Risks and Benefits. Front. Physiol..

[16] Morgan D.L., Allen D.G. (1999). Early events in stretch-induced muscle damage. J. Appl. Physiol..

[17] Torres R., Vasques J., Duarte J.A., Cabri J.M. (2010). Knee proprioception after exercise-induced muscle damage. Int. J. Sports Med..

[18] Bennett G.J., Liu G.K., Xiao W.H., Jin H.W., Siau C. (2011). Terminal arbor degeneration--a novel lesion produced by the antineoplastic agent paclitaxel. Eur. J. Neurosci..

[19] Kouzaki K., Nosaka K., Ochi E., Nakazato K. (2016). Increases in M-wave latency of biceps brachii after elbow flexor eccentric contractions in women. Eur. J. Appl. Physiol..

[20] Vincent J.A., Nardelli P., Gabriel H.M., Deardorff A.S., Cope T.C. (2015). Complex impairment of IA muscle proprioceptors following traumatic or neurotoxic injury. J. Anat..

[21] Vincent J.A., Wieczerzak K.B., Gabriel H.M., Nardelli P., Rich M.M., Cope T.C. (2016). A novel path to chronic proprioceptive disability with oxaliplatin: Distortion of sensory encoding. Neurobiol. Dis..

[22] Bullinger K.L., Nardelli P., Pinter M.J., Alvarez F.J., Cope T.C. (2011). Permanent central synaptic disconnection of proprioceptors after nerve injury and regeneration. II. Loss of functional connectivity with motoneurons. J. Neurophysiol..

[23] Alvarez F.J., Titus-Mitchell H.E., Bullinger K.L., Kraszpulski M., Nardelli P., Cope T.C. (2011). Permanent central synaptic disconnection of proprioceptors after nerve injury and regeneration. I. Loss of VGLUT1/IA synapses on motoneurons. J. Neurophysiol..

[24] Sonkodi B. (2021). Delayed Onset Muscle Soreness (DOMS): The Repeated Bout Effect and Chemotherapy-Induced Axonopathy May Help Explain the Dying-Back Mechanism in Amyotrophic Lateral Sclerosis and Other Neurodegenerative Diseases. Brain Sci.

[25] Thompson A.K., Mrachacz-Kersting N., Sinkjaer T., Andersen J.B. (2019). Modulation of soleus stretch reflexes during walking in people with chronic incomplete spinal cord injury. Exp. Brain Res..

[26] Corna S., Grasso M., Nardone A., Schieppati M. (1995). Selective depression of medium-latency leg and foot muscle responses to stretch by an alpha 2-agonist in humans. J. Physiol..

[27] Sinkjaer T., Andersen J.B., Nielsen J.F. (1996). Impaired stretch reflex and joint torque modulation during spastic gait in multiple sclerosis patients. J. Neurol..

[28] Schieppati M., Nardone A. (1997). Medium-latency stretch reflexes of foot and leg muscles analysed by cooling the lower limb in standing humans. J. Physiol..

[29] Nardone A., Schieppati M. (1998). Medium-latency response to muscle stretch in human lower limb: Estimation of conduction velocity of group II fibres and central delay. Neurosci. Lett..

[30] Sinkjaer T., Andersen J.B., Nielsen J.F., Hansen H.J. (1999). Soleus long-latency stretch reflexes during walking in healthy and spastic humans. Clin. Neurophysiol..

[31] Grey M.J., Ladouceur M., Andersen J.B., Nielsen J.B., Sinkjaer T. (2001). Group II muscle afferents probably contribute to the medium latency soleus stretch reflex during walking in humans. J. Physiol..

[32] Uysal H., Larsson L.E., Efendi H., Burke D., Ertekin C. (2009). Medium-latency reflex response of soleus elicited by peroneal nerve stimulation. Exp. Brain Res..

[33] Af Klint R., Mazzaro N., Nielsen J.B., Sinkjaer T., Grey M.J. (2010). Load rather than length sensitive feedback contributes to soleus muscle activity during human treadmill walking. J. Neurophysiol..

[34] Uysal H., Boyraz I., Yagcioglu S., Oktay F., Kafali P., Tonuk E. (2011). Ankle clonus and its relationship with the medium-latency reflex response of the soleus by peroneal nerve stimulation. J. Electromyogr. Kinesiol..

[35] Hjortskov N., Essendrop M., Skotte J., Fallentin N. (2005). The effect of delayed-onset muscle soreness on stretch reflexes in human low back muscles. Scand. J. Med. Sci. Sports.

[36] Bencke J., Naesborg H., Simonsen E.B., Klausen K. (2000). Motor pattern of the knee joint muscles during side-step cutting in European team handball. Influence on muscular co-ordination after an intervention study. Scand. J. Med. Sci. Sports.

[37] Horvath M., Fazekas G. (2003). Assessment of motor impairment with electromyography--the kinesiological EMG. Ideggyogy. Szle..

[38] Nosaka K., Tiidus P.M. (2008). Muscle Soreness and Damage and the Repeated-Bout Effect. Skeletal Muscle Damage and Repair.

[39] Proske U., Gandevia S.C. (2012). The proprioceptive senses: Their roles in signaling body shape, body position and movement, and muscle force. Physiol. Rev..

[40] Sonkodi B., Hortobágyi T. (2022). Amyotrophic lateral sclerosis and delayed onset muscle soreness in light of the impaired blink and stretch reflexes – watch out for Piezo2. Open Med..

[41] Balestra C., Duchateau J., Hainaut K. (1992). Effects of fatigue on the stretch reflex in a human muscle. Electroencephalogr. Clin. Neurophysiol..

[42] Duchateau J., Hainaut K. (1993). Behaviour of short and long latency reflexes in fatigued human muscles. J. Physiol..

[43] Suchyna T.M. (2017). Piezo channels and GsMTx4: Two milestones in our understanding of excitatory mechanosensitive channels and their role in pathology. Prog. Biophys. Mol. Biol..

[44] Bewick G.S., Banks R.W. (2021). Spindles are doin’ it for themselves: Glutamatergic autoexcitation in muscle spindles. J. Physiol..

[45] Sonkodi B., Resch M.D., Hortobágyi T. (2022). Is the Sex Difference a Clue to the Pathomechanism of Dry Eye Disease? Watch out for the NGF-TrkA-Piezo2 Signaling Axis and the Piezo2 Channelopathy. J. Mol. Neurosci..

[46] Chen T.J., Kukley M. (2020). Glutamate receptors and glutamatergic signalling in the peripheral nerves. Neural Regen Res..

[47] Spitzer S., Volbracht K., Lundgaard I., Karadottir R.T. (2016). Glutamate signalling: A multifaceted modulator of oligodendrocyte lineage cells in health and disease. Neuropharmacology.

[48] Kubo A., Koyama M., Tamura R., Takagishi Y., Murase S., Mizumura K. (2012). Absence of mechanical hyperalgesia after exercise (delayed onset muscle soreness) in neonatally capsaicin-treated rats. Neurosci. Res..

[49] Sufka K.J., Price D.D. (2002). Gate Control Theory Reconsidered. Brain Mind.

[50] Borghi S.M., Bussulo S.K.D., Pinho-Ribeiro F.A., Fattori V., Carvalho T.T., Rasquel-Oliveira F.S., Zaninelli T.H., Ferraz C.R., Casella A.M.B., Cunha F.Q. (2021). Intense Acute Swimming Induces Delayed-Onset Muscle Soreness Dependent on Spinal Cord Neuroinflammation. Front. Pharmacol..

